# Editorial: Sensitization and Desensitization in Organ Transplantation

**DOI:** 10.3389/fimmu.2021.784472

**Published:** 2021-10-14

**Authors:** Imran J. Anwar, Annette M. Jackson, Jayme E. Locke, Jean Kwun

**Affiliations:** ^1^ Duke Transplant Center, Department of Surgery, Duke University Medical Center, Durham, NC, United States; ^2^ Comprehensive Transplant Institute, University of Alabama at Birmingham, Birmingham, AL, United States

**Keywords:** sensitization, desensitization, transplantation, donor specific antibodies, antibody-mediated rejection (AMR)

Transplantation is the gold standard treatment for end-stage organ failure. Sensitization, the prior exposure to non-self human leukocyte antigen (HLA), is a major impediment to transplantation as it reduces the chances of finding a suitable donor and places individuals at heightened immunological risk, resulting in worse transplant outcomes. As such, there has been great interest in understanding the immunologic nature of sensitization and developing therapies to desensitize these patients and improve outcomes. This Research Topic, encompassing 14 contributions from several groups, aims at providing updates on various topics related to sensitization – current organ-specific practices regarding management of the sensitized patient, etiology of sensitization, desensitization approaches, and new tools available to study sensitization. The consequences and management of sensitization across transplant organ type varies dramatically and comprehensive reviews of the current management of sensitized patients in heart (Habal), lung (Young et al.), and vascularized composite allotransplantation (Moris and Cendales) are included in this special Research Topic edition.

Sensitization occurs through a variety of different modalities, namely prior transplantation, blood transfusions, and pregnancies. While those modalities lead to similar consequences, exposing individuals to non-self HLA, it is unknown whether the immunologic responses related to those exposures are similar across modalities. Nguyen et al. demonstrated, in a retrospective cohort of sensitized heart transplant candidates, desensitization protocols were less efficacious in women, particularly women sensitized due to prior pregnancies. This suggests that not every sensitization event is equal; rather, there exists key differences in the immunologic response to non-self HLA exposure, leading to heterogenous outcomes regarding desensitization. The unique “immunologic paradox” of pregnancy as a sensitization event is thoroughly reviewed by Nellore et al. with an emphasis on memory B cell formation and its correlation with pregnancy-related sensitization.

Screening for circulating HLA antibodies is standard of care prior to transplantation as part of the risk assessment process to optimize donor selection and transplant outcomes. HLA-specific Luminex assays permit sensitive antibody screening across hundreds of HLA alleles. However, these assays are seldom used in research given their prohibitive cost and unavailability for MHC molecules relevant to common animal transplant models. Song et al. described a novel set of techniques that allow for specific measurements of major histocompatibility complex (MHC) sensitization across various species. These tools offer the promise of in-depth characterization of MHC mismatches in animal models commonly used to study sensitization in transplantation.

Various approaches have been proposed to improve organ availability and outcomes for sensitized patients. Donor allocation policies have been modified in recent years to reduce the gap between transplantation rates between non-sensitized and sensitized individuals. The Kidney Allocation System (KAS) was implemented in the United States in 2014 to facilitate kidney transplantation in highly sensitized individuals by increasing the pool of donors and giving priority to highly sensitized individuals, resulting in increased transplant rates. The Eurotransplant Acceptable Match (AM) program was founded in 1989 with the goal of increasing transplantation rates in highly sensitized patients in the region. Their matching algorithm takes into account the recipient’s HLA antigens in combination with pre-defined acceptable antigen mismatches. Heidt et al. summarized the AM program experience and showed high organ offer (80%) and similar outcomes compared to non-sensitized patients regarding rejection and long-term graft survival. The use of predefined acceptable antigen mismatches when matching sensitized individuals is thus a promising approach to increase transplant rate in allocation systems.

Despite efforts to optimize allocation systems, the most highly sensitized individuals (CPRA > 99.9%) remain on the waitlist longer than non-sensitized patients and experience greater mortality and morbidity. Desensitization has long been advocated as a mean to achieve transplantation in this high-risk population. Schinstock et al. provided thoughtful guidance regarding which patients might benefit from desensitization prior to kidney transplantation in the current KAS era.

A myriad of pharmacologic and non-pharmacologic treatments have been brought forward as desensitization regimens and were thoroughly reviewed in Choi et al.. However, no desensitization therapy has yet solved the barriers to transplantation faced by highly sensitized patients. Kumar and Locke succinctly describe recent advances in the desensitization field, including novel promising pharmacologic approaches and development of novel assays that allow for precise HLA epitope matching. Plasmapheresis remains the mainstay of many protocols aiming at removing circulating donor-specific antibodies (DSA). Manook et al. investigate modulation of the neonatal Fc receptor (FcRn) as a pharmacological alternative to plasmapheresis. Inhibition of FcRn successfully lowers donor-specific antibody (DSA) titers, as shown by reduced T-cell and B-cell crossmatch, in a sensitized non-human primate model. However, the therapy did not prevent rapid DSA rise post kidney transplant or prolong graft survival. Nevertheless, inhibition of FcRn does show potential to reduce DSA in a pre-transplant setting with fewer side effects compared to plasmapheresis.

Plasma cells, the main producers of antibodies, are an obvious target to manage the humoral response in sensitized patients. However, conventional therapies fail to deplete plasma cells given their lack of CD20 expression. CD38-targeting immunotherapies have played an increasingly important role as plasma cell-depletion agents in myeloma treatments. Joher et al. provided a thorough review of those immunotherapies and their possible application to organ transplantation. In this review, it was astutely noted that anti-CD38 therapies induce immune deviation, a phenomenon thought to enhance host-anti-tumor immune responses but with possible deleterious consequences in organ transplantation. Depletion of CD38+ immunosuppressive cells across multiple lineages may be responsible for the elevated antiviral protection as well as elevated alloreactive functional responses.

Sensitized individuals are at a higher risk of developing antibody-mediated rejection (AMR) compared to non-sensitized patients. Histopathology is central to the diagnosis of AMR and the Banff 2019 classification system is commonly used to diagnose AMR. Despite its widespread use, this classification system contains several limitations such as lack of reproducibility and is based on biopsy findings without incorporating clinical features. In her review article, Dr. Cornell elegantly reviewed the most recent Banff classification for AMR, focusing on how it relates to patients undergoing desensitization or undergoing incompatible transplants and offered a possible framework for considering allograft injury associated with DSA as a continuum.

Finally, strategies to induce tolerance would minimize sensitization and abrogate the need for desensitization therapies and life-long immunosuppression. Cellular therapies have been explored since the 1950s to induce non-responsiveness to transplanted allografts. Despite various trials, the balance between efficacy and safety has limited broader implementation. Shaw et al. thoroughly reviewed the history of cellular therapies as they pertain to solid organ transplantation and contrast their salutary effects with their potential to induce sensitization.

To summarize, the collection of review and research articles presented under this Research Topic provides a comprehensive set of information on advances in sensitization and desensitization in solid organ transplantation. Further understandings of the etiology, mechanisms, and consequences of sensitization should provide chances to develop novel therapies to facilitate transplantation in the highly sensitized population.

## Author Contributions

IA, AJ, JL, and JK participated in writing and reviewing the manuscript.

## Conflict of Interest

The authors declare that the research was conducted in the absence of any commercial or financial relationships that could be construed as a potential conflict of interest.

## Publisher’s Note

All claims expressed in this article are solely those of the authors and do not necessarily represent those of their affiliated organizations, or those of the publisher, the editors and the reviewers. Any product that may be evaluated in this article, or claim that may be made by its manufacturer, is not guaranteed or endorsed by the publisher.

